# A randomised cross-over study assessing the “blue pyjama syndrome” in major depressive episode

**DOI:** 10.1038/s41598-017-02411-x

**Published:** 2017-06-01

**Authors:** Hélèna Delmas, Jean-Marie Batail, Bruno Falissard, Gabriel Robert, Maxence Rangé, Stéphane Brousse, Jacques Soulabaille, Dominique Drapier, Florian Naudet

**Affiliations:** 1 Academic Psychiatry Department, Centre Hospitalier Guillaume Régnier, Rennes, France; 2 EA 4712 Behavior and Basal Ganglia, CHU Rennes, Rennes 1 University, Rennes, France; 3 CESP, Univ.Paris-Sud, Université Paris-Saclay, UVSQ, INSERM U1178, Paris, France; 4 INSERM CIC-P 1414, Clinical Investigation Center, CHU Rennes, Rennes 1 University, Rennes, France; 5 Meta-Research Innovation Center at Stanford (METRICS), Stanford School of Medicine, Palo Alto, CA 94304 USA

## Abstract

This paper introduces a “blue pyjama syndrome” (whereby wearing hospital pyjamas results in an exaggerated impression of severity). We performed a 5-day, prospective, randomized, cross-over study in a French mood disorder unit for inpatients. At Day 1 (D1) and Day 5 (D5), two 5-minute video interviews were recorded with patients in pyjamas or in day clothes (the sequence was randomly allocated). Psychiatrists unaware of the study objective assessed the videos and scored their clinical global impressions (CGI, with scores ranging from 1 to 7). Of 30 participants with major depressive episode selected for inclusion, 26 participants (69% women) provided useable data for an evaluation by 10 psychiatrists. Pyjamas significantly increased the psychiatrists’ CGI ratings of disease severity by 0·65 [0·27; 1·02] points. The psychiatrists’ global impressions also rated patients as significantly less severe at D5 in comparison with D1 by −0·66 [−1·03; −0·29] points. The “blue pyjama syndrome” is in the same order of magnitude as the difference observed after a week of hospitalisation. This potentially calls into question the reliability and validity of observer ratings of depression.

## Introduction

Social imagination often pictures the psychiatrist as a doctor with a kind of mystical power enabling direct perception of the deep, closed, mysterious human psyche. More modestly, in their day-to-day practice, psychiatrists try to apply the crucial “art of understanding”^1^ to address the specific needs of patients who are suffering from psychiatric illness. The clinical interview is a key component of psychiatric care, since it aims both to gain the confidence of patients and to gather critical information. It provides a clinical impression which is the cornerstone of both diagnostic and therapeutic reasoning. But like all social interactions, the impression resulting from a given interview can be misled by subtle, unconsciously perceived cues such as the presentability of the patient.

In some countries, such as France, the use of pyjamas for inpatients (mainly to prevent suicide and/or escape) is one possibly stigmatizing aspect of the management of inpatients suffering from major depressive episodes (MDE)^2^. Wearing a uniform was part of the norm in early “asylum care”. After World War Two, and concomitantly with the discovery of new and effective drugs, psychiatric care profoundly altered. The use of uniforms was gradually phased out. Nevertheless, many acute psychiatric in-patient units maintained a policy of placing newly admitted patients, both voluntary and involuntary, in night attire and withholding their day clothes^3^. Despite the absence of evidence for its usefulness, it is still a common practice in most French psychiatric hospitals, raising ethical debates from within and outside of the mental health profession^4^. Without entering further into these debates, our clinical intuition was that the presentation in blue pyjamas (i.e. blue scrubs) resulted in an exaggerated impression of severity. We call this possible bias the “blue pyjama syndrome” modelled on the “white coat syndrome” in the context of hypertension^5^.

### Aims of the study

In this paper, we report on a study on the reliability of assessments in psychiatry aiming to better understand subjective measurement processes in MDE, by exploring the existence of the “blue pyjama syndrome”, and quantifying its impact.

## Material and Methods

### Eligibility

The study was conducted in the mood disorder unit of the Adult Psychiatry University Department of Rennes France. Adult inpatients with mood disorders and a current MDE (as defined by the DSM IV^6^), diagnosed using the Mini-International Neuropsychiatric Interview^7^ (MINI) and who were able to understand the study design were eligible. Patients under guardianship or trusteeship, or suffering from schizophrenia or with a medical need to be in pyjamas were not included in the study. Eligible patients were each given an information letter describing the study design (see https://osf.io/24r7k/ for the detailed letter in French). All participants provided written informed consent to take part in the trial. All procedures contributing to this work are in accordance with the relevant guidelines and regulations. The protocol was approved by the local committee (Comité d’éthique du CHU de Rennes) of Rennes, France, on 05 March 2015 (Avis n° 15.15, see https://osf.io/vpu8w/).

### Trial design, randomisation and masking

This was a 5-day, prospective, randomized, cross-over study performed in a single centre comparing presentation in pyjamas to presentation in day clothes. After an initial assessment of clinical (medical history, MINI) and socio-demographic data, all patients were randomly assigned to one of two sequences of two assessments: (1) Assessment in pyjamas at day one (D1) and in day clothes at day five (D5) or (2) Assessment in day clothes at D1 and in pyjamas at D5. The period of 5 days was chosen for practical reasons. This 5-day time-lapse was intended 1/ to avoid any constraint for patients (for example the need to come back to the hospital after a hospitalization), 2/ to minimize heterogeneity and 3/ to limit missing data. The investigator used closed envelopes provided by the methodologist containing the randomization status for each patient, in accordance with a computer-generated randomisation list with a 1:1 ratio.

**Figure 1:**
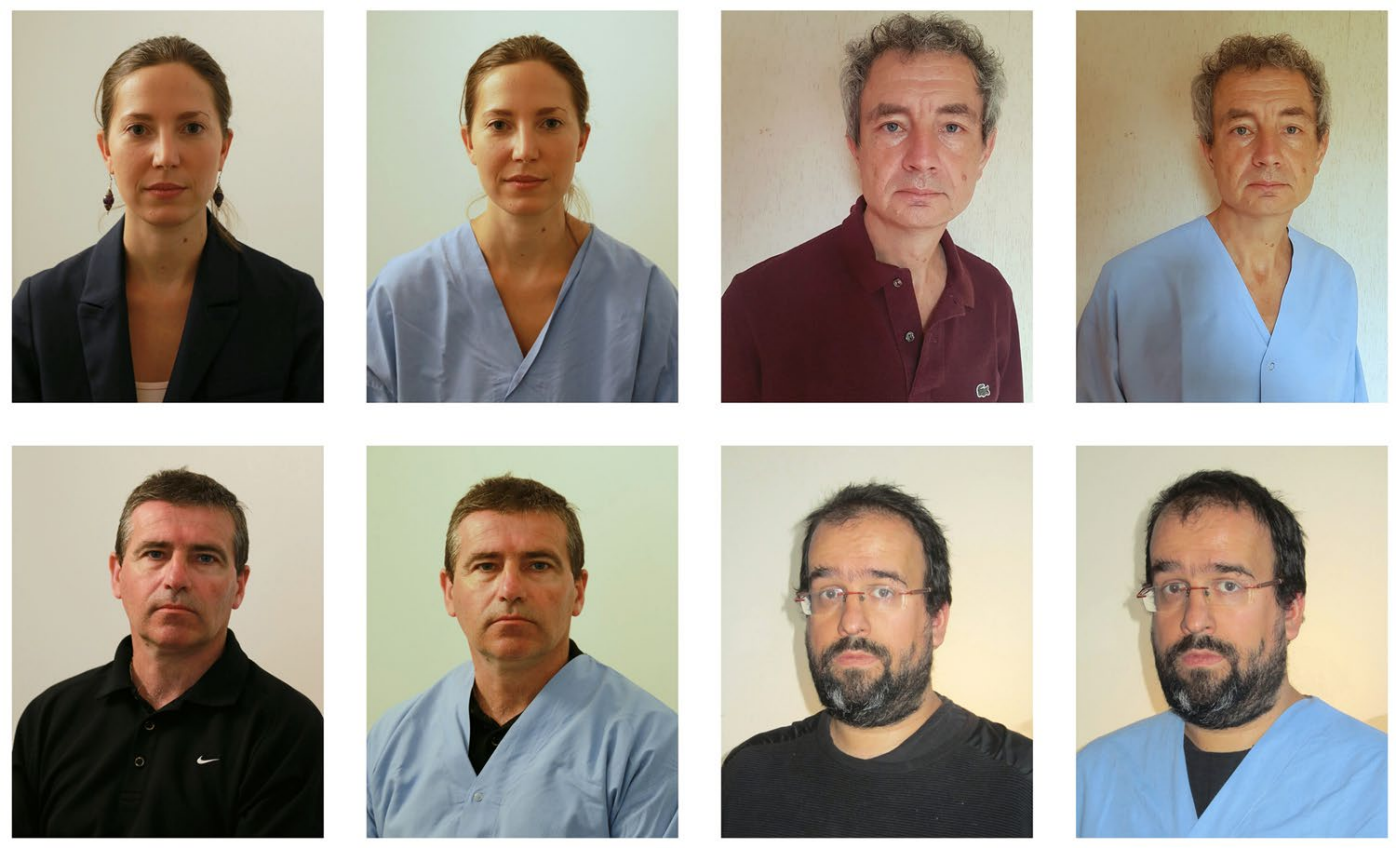
Four members of the team in pyjamas and in day clothes.

D1 and D5 assessments were identical and based on (1) the video recording of a 5-minute standardised interview (details are described elsewhere^8^) and (2) the Beck Depression Inventory (BDI)^9^, a self report inventory. We used our hospital blue pyjamas as the standardised intervention (as illustrated by our team in Fig. 1) and patients used their day clothes in the control condition. All interviews were performed by the same investigator.

10 psychiatrists were recruited to participate in the study. Psychiatrists from the same team as the investigators were not included in order 1/ to avoid them assessing their own patients and 2/ to be sure that they would not guess the study design. After collection of their socio-demographic characteristics, each psychiatrist was asked to rate 10 videos (or in the case of 4 psychiatrists, 11). The videos were randomly assigned to each psychiatrist using the following rules: 1/ Each video was to be seen by two psychiatrists; 2/ Each psychiatrist was to see an imbalanced distribution (3:7 or 4:7 OR 7:3 or 7:4) of patients in pyjamas or in day clothes (this artifice was to avoid awakening the psychiatrists’ attention to the study objective); 3/ Each psychiatrist was to see only one video for each patient. This second computer-generated randomisation list was prepared by the methodologist and sent to the investigator who prepared the videos for each psychiatrist.

Each psychiatrist was instructed in the scoring of the Clinical Global Impressions scale (CGI), was asked to read a scoring guide, as described previously^8^ and for each video was to answer the following question “Considering your overall clinical experience with this type of patient, how mentally ill is the patient at this time?”.

The CGI scoring was performed using a Visual Analogue Scale (VAS) in order to collect a continuous outcome that would be easier to handle in our statistical models than the usual discrete outcomes collected with the traditional CGI. The VAS was graduated in order to present the 7 usual categories in the CGI. The use of VAS is common in clinical research and in psychiatry in particular, it has already been used and has demonstrated good inter-rater reliability and good correlation with other depression scales^10–12^.

Importantly, the psychiatrists were not aware of the study objective (deceptive design) and were told that the purpose of this study was to improve the CGI scale with the use of a VAS. They were debriefed after study completion as to the exact objective of the study.

The study was registered with the Open Science Framework on 18 May 2016 as soon we were aware that this framework offered the possibility of preregistration with an embargo period (registration number: osf.io/gcw9e; see https://osf.io/24r7k/ for the detailed protocol in French). Indeed, because the study design involved deceit towards psychiatrists, we did not want a preregistration to be publicly available before data collection was complete.

### Statistical Analysis

The principal outcome was the difference in CGI score between the pyjama condition and the day clothes condition. Because the study was a cross-over study in the course of a one-week hospitalisation (the unit hospitalises one week at a time, which can be renewed), it was possible to assess the pre-post CGI difference as a secondary outcome. We planned this assessment in order to put the “pyjama effect” into perspective with the “one week of hospitalisation effect”. To take into account the correlated nature of the data gathered, this analysis was performed using a mixed model with the CGI score as the dependent variable and the following explanatory variables: 1/ pyjamas (yes/no) and 2/ hospitalisation (D1 or D5). This mixed model was performed with the “patient” and the “psychiatrist” factors specified as random effects. The results of this model are the effect of the explanatory variable expressed as CGI scores with their 95% confidence interval. Inter-rater agreement was assessed using the intraclass correlation coefficient (ICC) as defined by the ratio of the inter-patient variance to the sum of the inter-patient variance, the inter-rater variance and the residual variance^13^.

Finally, in order to explore whether any pyjama effect evidenced on the CGI was due to a pyjama effect in the clinicians’ evaluations or whether pyjamas had a genuine effect on patients’ mood, we analysed BDI scores as another secondary outcome. We also analysed the D1-D5 difference as assessed on the BDI. These analyses were performed using a pairwise t-test (two-tailed, P < 0.05).

Descriptive data were summarized numerically, with mean (+/−standard deviation) for quantitative data and numbers (percentages) for categorical data. All the statistical analyses were performed with R (R Development Core Team, version 3.2.1), with the library lme4^14^.

Because of the complex nature of the design used here, we calculated the number of subjects that would be required for a univariate analysis and considered that it would be sufficient for our multivariate analysis. We hypothesized that the mean severity as measured by the CGI VAS would be 5 points (+/−1 points) in pyjama condition and 4 points (+/−1 points) in day clothes. On the basis of these hypotheses, the number of subjects required was 26 with 2 videos for each subject with the alpha and beta risks set at 0.05. To avoid a saturation of fatigue effect among observers, which would have increased measurement error, we decided to ask observers to participate no more than an estimated 2 hours (including instructions and assessment of the videos). We therefore decided that 10 psychiatrists would assess 10–11 videos.

### Sensitivity analysis

Following a comment made during the peer review process, we performed a post-hoc sensitivity analysis to explore whether there was an interaction between the effect of the hospitalisation on the rater’s perception and the effect of pyjamas on his/her perception. We performed the same model as in our pre-specified analysis but we added an interaction term. An analysis of variance, and a model fit criterion (Akaïke’s Information Criterion (AIC)) were used to compare the two models.

### Role of the funding source

The sponsor had no role concerning the preparation, review, or approval of the manuscript.

## Results

### Patients and psychiatrists

**Figure 2:**
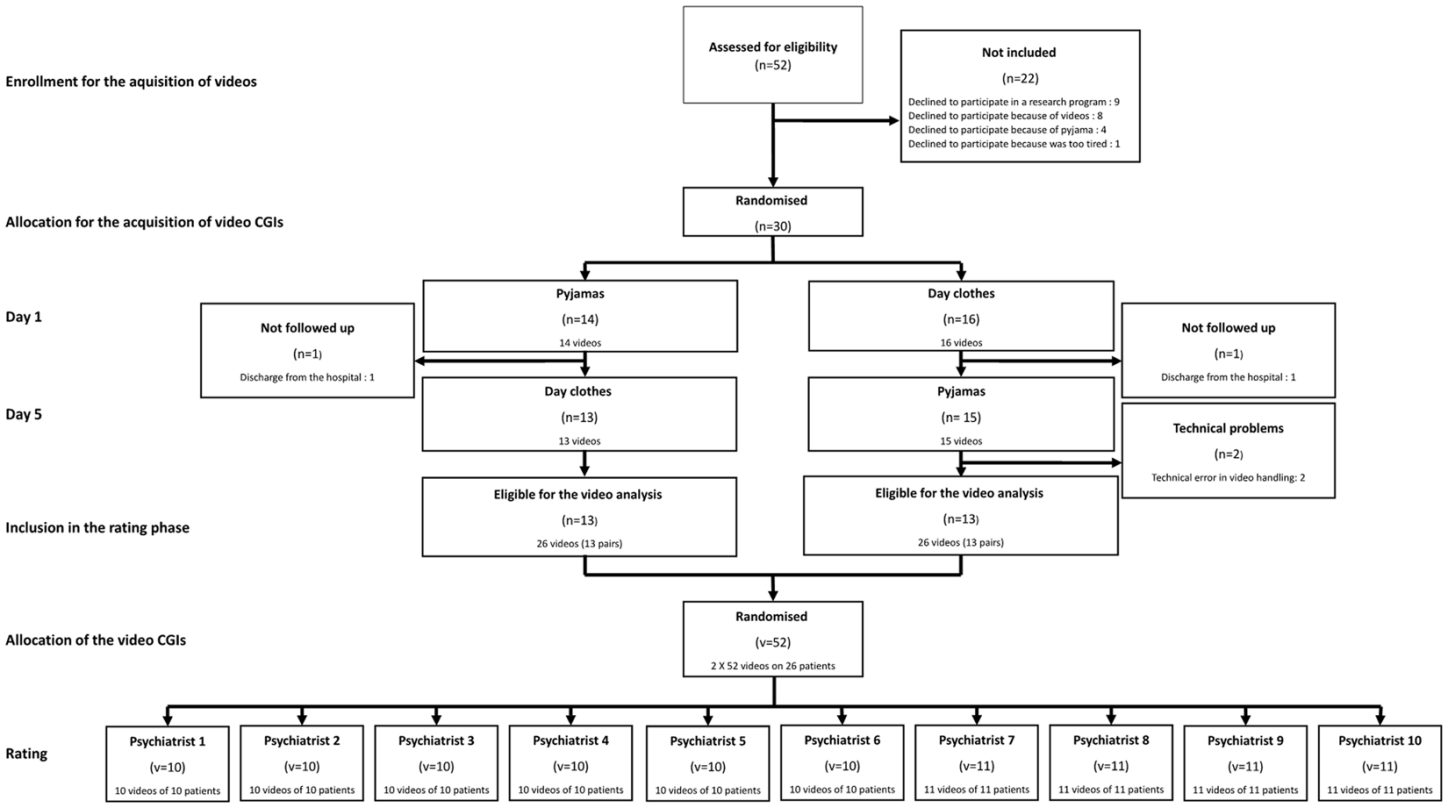
Study flowchart.

From May 2015 to June 2016, a total of 52 eligible patients were screened. 22 patients refused to participate in the study. For 2 patients, the second video was not useable due to technical problems and 2 patients left the hospital before the D5 assessment (it was not possible to film them after discharge because the video recorder had to stay in the hospital). Because these problems were independent from the study, it was decided to replace these patients. Therefore 26 patients completed the 2 video assessments (see Fig. 2). The clinical and demographic characteristics of these patients are presented in Table 1. From June 2016 to July 2016, a total of 11 eligible psychiatrists were identified and 10 agreed to participate in the study (one had no time for this research). They were aged of 43+/−9 years and 5 (50%) were women.

**Table 1:** Demographic and Clinical Characteristics of the 26 Patients with Major Depressive Episode (MDE). For all results, data are summarized numerically, with mean (+/−Standard Deviation) for quantitative outcomes and numbers (percentage) for categorical outcomes.

	Values
**Demographic characteristics**	
Age	51+/−13
Female gender	18 (69%)
Occupation	
*Employed*	5 (19%)
*Unemployed*	2 (8%)
*Student*	1 (4%)
*Retired*	5 (19%)
*Long term sick leave*	13 (50%)
Married or living with a partner	18 (69%)
Has children	21 (81%)
**Clinical characteristics**	
Duration of the current MDE (months)	25+/−29
Type of mood disorder	
*Major depressive disorder (single episode)*	7 (27%)
*Major depressive disorder (recurrent)*	13 (50%)
*Bipolar disorder*	6 (23%)
History of suicide attempt	13 (50%)
Pyjama use in previous hospitalization	6 (24%)^†^
**Treatments in use at admission**	
Antidepressants	21 (81%)
Mood stabilizer	10 (38%)
Antipsychotics	10 (38%)
Sedative antipsychotics	6 (23%)
**Evolution during hospitalisation**	
Change of background treatment	6 (23%)
Antidepressants	
*Instatement*	3 (12%)
*Removal*	2 (8%)
Antipsychotics	
*Instatement*	1 (4%)
*Removal*	3 (12%)
Received repetitive Transcranial Magnetic Stimulation	4 (15%)
Received Electroconvulsive Therapy	1 (4%)
BDI at D1	19.36+/−8.01^††^
BDI at D5	12.50+/−7.78^†††^

D1: Day 1. D5: Day 5. BDI: Beck Depression Inventory. ^†^1 missing data. ^††^For one patient, 3 items in the BDI were missing and replaced by the mean score on the scale. ^†††^For two patients, 3 items in the BDI were missing and replaced by the mean score on the scale.

### Primary outcome: analysis of the CGI

**Figure 3:**
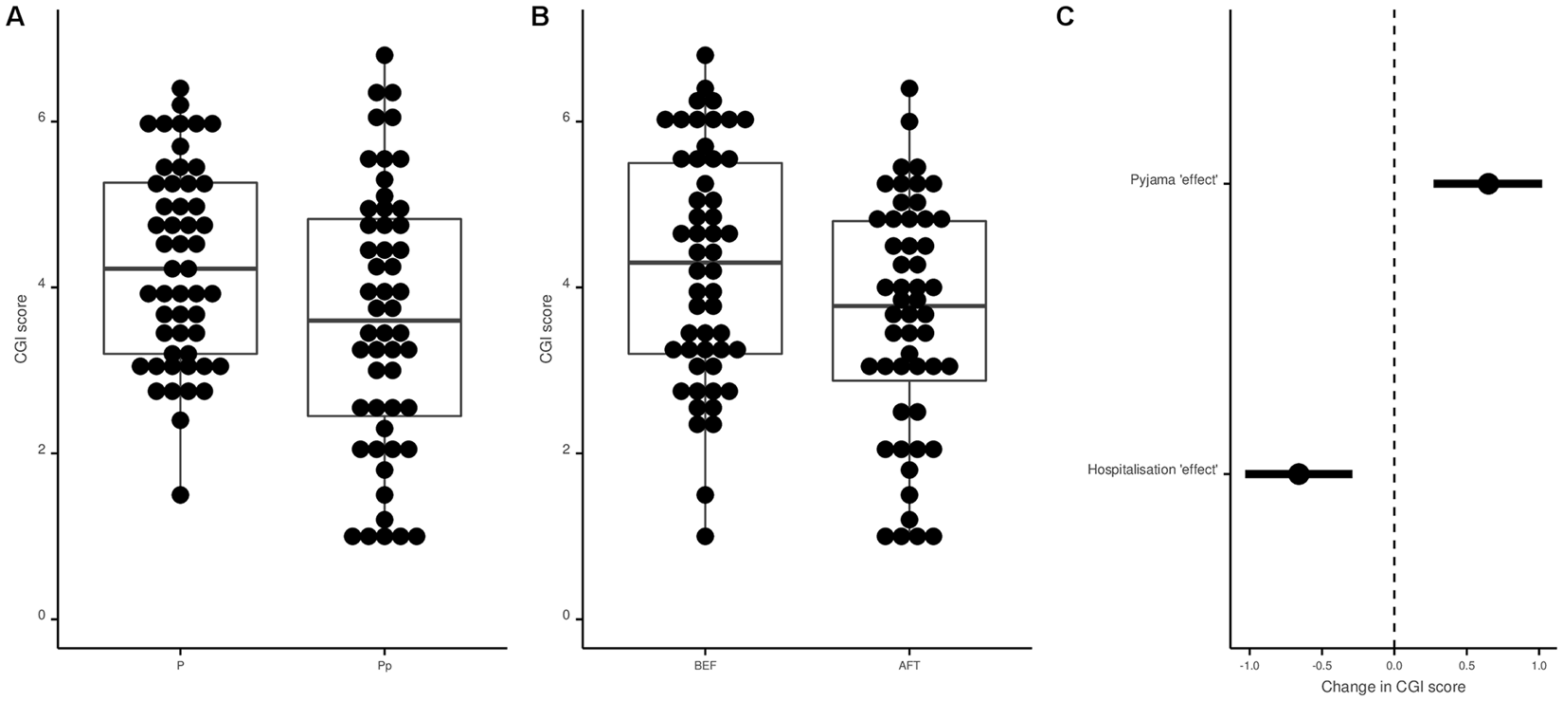
Pyjama and hospitalization effects on Clinical Global Impressions (CGI). Panel A: Distribution of CGI scores in the day clothes and pyjama conditions. Data are presented for descriptive purpose only. The dots represent each value for each patient (a given patient has 2 values in each condition). Panel B: Distribution of CGI scores at Day 1 and Day 5. Data are presented for descriptive purposes only. The dots represent each value for each patient (a given patient has 2 values in each condition). Panel C: CGI analysis; Forest plot of coefficients and their 95% confidence interval observed with the mixed model (mixed model performed with the “patient” and the “psychiatrist” factors specified as random effects).

Figure 3 presents the results concerning the CGI: pyjamas significantly increased the psychiatrists’ global impression of severity by 0·65 [0·27; 1·02] points (p = 0.001). The psychiatrists’ global impressions significantly rated patients as less severe at D5 in comparison with D1 by −0·66 [−1·03; −0·29] (p < 0.001). The ICC was 0.51. Data to reproduce this analysis are available here (https://osf.io/kh28j/) and the corresponding code is available here (https://osf.io/e6zqu/). After the study, all psychiatrists who rated the videos were debriefed, and none had guessed the objective of the study.

### Secondary outcome

**Figure 4:**
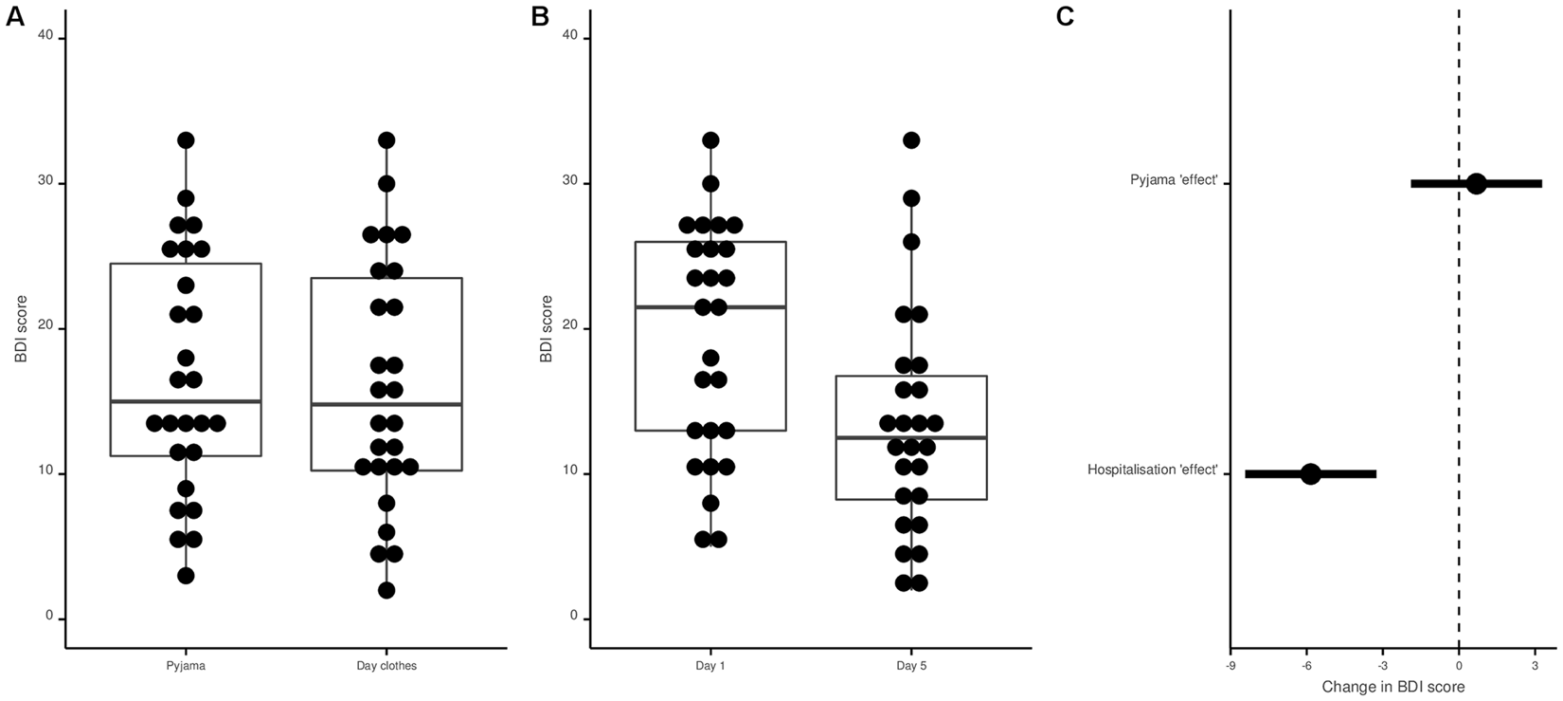
Pyjama and hospitalization effects on the Beck Depression Inventory (BDI). Panel A: Distribution of BDI scores in the day clothes and pyjama conditions. Panel B: Distribution of BDI scores at Day 1 and Day 5. Panel C: BDI analysis; forest plot of coefficients and their 95% confidence interval observed with a mixed model (mixed model performed with the “patient” factors specified as random effect).

No difference was found in the analysis of the BDI self-report inventory scores between the pyjama and the day clothes conditions (mean difference = 0·69+/−8·88; p = 0·69) while the score at D1 was 5.84 (+/−6·62) points higher than the score observed at D5 (p < 0·001). These results were also observed in the multivariate model (Fig. 4). Data to reproduce these analyses are available here (https://osf.io/jdmtq/) and the code is available here (https://osf.io/e6zqu/).

### Sensitivity analysis

**Figure 5:**
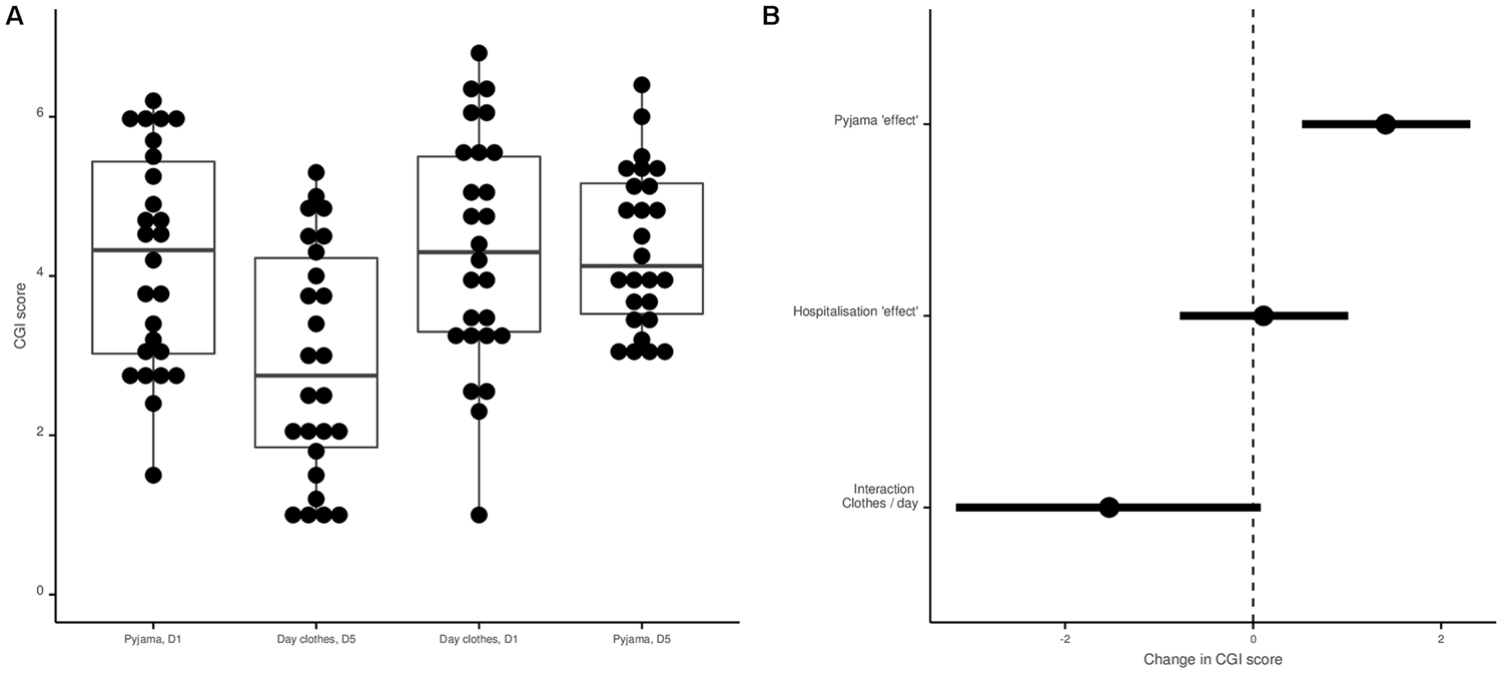
Pyjama and hospitalization effects and interaction on Clinical Global Impressions (CGI). Panel A: Distribution of CGI scores at Day 1 (D1) and Day 5 (D5) in the day clothes and pyjama conditions. Data are presented for descriptive purposes only. The dots represent each value for each patient (a given patient has 2 values in each condition). Panel B: CGI analysis; Forest plot of coefficients and their 95% confidence interval observed with the mixed model including an interaction (mixed model performed with the “patient” and the “psychiatrist” factors specified as random effects). Negative values for the interaction term (Clothes/days) means that the positive effect perceived after 5 days of hospitalization is more marked when patients are in day clothes than in pyjamas.

Figure 5 presents the results of the post-hoc sensitivity analysis. In this model, pyjamas significantly increased the psychiatrists’ global impression of severity by 1·42 [0·52; 2·31] points (p = 0.004). In addition, the psychiatrists’ global impressions did not necessarily rate patients as less severe at D5 in comparison with D1 (difference of 0·11 [−0·78; 1·01], p = 0.803). Although the result did not reach significance, the assessment rather depended on the patient’s attire at Day 5 (interaction of 1·53 [−0·08; 3·15], p = 0.075). The AIC criterion suggested that this model was slightly more parsimonious than our pre-specified model (AIC of 338.42 versus 339.93) although this better fit did not reach statistical significance (p-value = 0.06). Data to reproduce this analysis are available here (https://osf.io/kh28j/) and the corresponding code is available here (https://osf.io/e6zqu/).

## Discussion

### Statement of principal findings

This study confirmed our first intuition concerning the “blue pyjama syndrome”. While the presentation in pyjamas did not affect patients’ self-report of depression severity, it affected the clinicians’ subjective impressions since they rated higher levels of severity for patients in pyjamas. This difference was in the same order of magnitude as the improvement observed after one week of hospitalisation also confirmed by the self-report of depression severity.

Interestingly, although not statistically significant, the post-hoc sensitivity analysis suggested that this pre-post improvement was mostly perceived by psychiatrists when the patient was not dressed in pyjamas at Day 5. This result suggests stimulating hypotheses. It could be that “the blue pyjama syndrome” is more marked in less severe clinical presentations. Alternatively, it is possible that the improvement observed during hospitalization might translate into changes in presentation that could be easy to identify but are masked by the presentation in blue pyjamas. Further research is needed to confirm this possible interaction and disentangle the possible interpretations of this result.

### Strengths and weaknesses of the study

While the pre-post difference could be due to various factors such as regression to the mean, spontaneous improvement, placebo effect, or, indeed effects of the different therapeutic changes that occurred during the one-week hospitalization^15^, none of these factors are likely to have confounded our estimation of the ‘blue pyjama syndrome’ in the context of this randomized controlled trial. The inter-rater agreement was acceptable in our study. A value of 0.50 is usually considered as being fair^16^. Nevertheless, certain limitations can be taken into account with this specific design. First, any experiment is somewhat artificial, and even if the video CGI 1/ is validated, 2/ is considered as holistic in its approach and 3/ enables a phenomenological understanding of a given patient^9^, the 5-minute videoed interview cannot recreate a typical clinical encounter where the clinical impressions are built up session after session, providing material for a more comprehensive understanding. Moreover, a video could induce artefactual attitudes or coping strategies that could be different from those in a direct face-to-face interview. Furthermore, when only a brief videotaped interview is available the clinician could be more likely to make use of non-verbal information to a larger degree than in a normal interview setting. Specifically, using CGIs, which are not focused on symptoms but on overall functioning, may have limited use in an inpatient setting where more detailed information is usually required. On the other hand, at least in France, most clinicians do not use depression rating scales for their day-to-day clinical practice and rely rather on their impressions. For this specific reason, we think that this choice was relevant. In addition, a substantial number of participants were not included in the study because they refused to participate (mainly because they were not comfortable with the idea of being videotaped).

The single ward location and the limited external validity in relation to other settings are two limitations that should be taken into account. Indeed, all participants were recruited from the same unit. In this unit patients 1/ are typically admitted for one or two weeks, 2/ are not under compulsory hospitalisation and 3/ are always in day clothes. Therefore, the patients included could represent an overly selected and homogenous subgroup of depressed patients. One might hypothesise that an opposite trend to the “blue pyjama syndrome” might be observed in patients from a different population, such as, for example, very severely depressed patients with extreme self-neglect. In this case, the presentation in clean blue pyjamas could bias the psychiatrist’s opinion toward an underestimation of the current episode severity. Despite the artificial situation of this randomised study, it might in fact be appropriate for psychiatrists to use diagnostic information from a patient’s attire. For instance, since pyjamas are often prescribed to patients because of risk of suicide or self-harm, the use of pyjamas might in fact be related to the clinical state and would probably be relevant to consider for clinicians, especially when the only information available is a short (5 min.) videotaped interview. In other words, patients in pyjamas are likely to be seen as more severely ill because it suggests that they are too ill to look after their appearance and maintain a daily routine, or because they are severely ill or at significant risk. This might sometimes be true in a real life setting but it was nonetheless not the case in our study. Finally, the sample size in our study is small. The wide confidence intervals observed in the post-hoc sensitivity analysis suggest that the study might be underpowered to provide a refined picture of the “blue pyjama” syndrome. Therefore, our results should not be over-interpreted and, as in most studies, the main interest is rather to invite reflection among clinicians. Clinicians should be aware of this possible bias related to their assessment of a given patient’s severity.

### Results in the context of the “pyjama literature”

Numerous studies have explored the possibility of a “rater bias” in randomized controlled trials. These studies explored whether blind raters were able to identify drug vs. placebo treatment in proportions that exceeded chance, for instance, because of adverse events^17, 18^ and how this could impact study outcomes^15, 19–24^. There is also a large body of literature on stigma in general, in the neurosciences^25^ and in psychiatry^26^. But to our knowledge, there is no literature about the bias resulting from the subjective perception of patients’ appearance nor specifically on the use of pyjamas.

The literature on pyjamas is indeed very sparse. A review of the PubMed database found 27 references with the “pyjama OR pajama” as keywords: only two references^3, 27^ addressed the use of pyjamas in psychiatric hospitals, and neither was experimental. The first one is a one-page paper published in 1982 that is no longer accessible, even by communicating with the author. The second is a critical analysis of this practice focused on enforced pyjama wearing^3^. Along these lines, Richard Lakeman suggests that this practice might be unequivocally incompatible with notions of patient-perceived recovery, and remarks that there is no evidence to suggest that it could contribute to clinical recovery. Precisely, and bearing in mind the limitations we have raised, our data suggests that the use of pyjamas may interfere with clinicians’ judgment about recovery. We found no epidemiological data about their use internationally. Further searches with the keyword “clothing” found a few references focused on sociological descriptions of the effect of nurses wearing street clothes in place of uniforms^28^, but no reference about patients wearing pyjamas. We are thus confident that we are the first to provide experimental data in “the pyjama literature” and encourage efforts to replicate this finding, including the use of alternative and more naturalistic designs such as observational studies.

Interestingly, the “blue pyjama syndrome” tackles a very crucial issue in the field of therapeutic research. There is indeed considerable debate about the small although statistically significant differences^29^ that are usually observed between antidepressants and placebos in MDE. Nonetheless, there is still no consensus and no convincing data to establish whether the differences are clinically meaningful or not^30, 31^. Let us take a concrete example. A recent meta-analysis of vortioxetine (the newest antidepressant) versus placebo, using the CGI, found a statistically significant drug-placebo disparity of 0.55 points for the 20 mg dose (smaller differences were observed for other doses)^32^. The efficacy of vortioxetine is thus based on evidence that is in the same order of magnitude as that for our “blue pyjama syndrome”.

### Perspectives

Of course, we used the term “blue pyjama syndrome” to be thought-provoking, not to claim that we have discovered a genuinely new psychiatric “syndrome”. In fact, the interest lies not in a study of pyjama-wearing per se, but in what our study says about the reliability and validity of observer ratings for depression. It indicates that ratings of the severity of depression are liable to be influenced by superficial factors concerning the patient’s attire that do not necessarily have any relationship with the severity of the condition. We can imagine that if such ratings are affected by what the patient is wearing, they will also be affected by the patient’s background and environment and other factors that may have no relationship with the condition that is supposed to be the subject of the assessment.

The controversial Roshenam experiment^33^, is an emblematic and historical example addressing the issue of reliability in psychiatric evaluation. Roshenam and other pseudo-patients gained admission to psychiatric hospitals by briefly reporting that that they had been hearing voices. After admission, they no longer reported symptoms and behaved as they ‘normally’ would. Despite this, many were treated as inpatients for substantial periods of time. Roshenam suggested that “the hospital itself imposes a special environment in which the meaning of behavior could easily be misunderstood”. Of course, our study is not as disruptive and provocative, but, in the same spirit, it questions whether clinicians and researchers are really able to rate the severity of patient condition in an unbiased, objective manner that reflects the real facts about the condition itself, or whether they are making a judgement based on preconceptions that probably reflect more about their own beliefs than the condition of the patient. This opens onto the complex issue of how we make judgements and produce quantitative data in psychiatry, and what it might really mean when we do so^34, 35^.

## References

[1] Shea, S. C. Psychiatric Interviewing: The Art of Understanding: a Practical Guide for Psychiatrists, Psychologists, Counselors, Social Workers, Nurses, and Other Mental Health Professionals. (Saunders, 1998).

[2] Giloux N (2016). Le pyjama hospitalier. Santé Mentale.

[3] Lakeman, R. Leave your dignity, identity, and day clothes at the door: the persistence of pyjama therapy in an age of recovery and evidence-based practice. *Issues Ment Health Nurs* **32**, 479–482, doi:10.3109/01612840.2011.579690 (2011). 10.3109/01612840.2011.579690 21736473

[4] Hazan, A. Rapport thématique du contrôleur général des lieux de privation de liberté «Isolement et contention dans les établissements de santé mentale», http://www.cglpl.fr/2016/isolement-et-contention-dans-les-etablissements-de-sante-mentale/ (2016).

[5] Kleinert HD (1984). What is the value of home blood pressure measurement in patients with mild hypertension?. Hypertension.

[6] American Pyschiatric Association. Diagnostic and Statistical Manual of Mental Disorders, Fourth Edition: DSM-IV-TR®. (American Psychiatric Association, 2000).

[7] Sheehan, D. V. *et al*. The Mini-International Neuropsychiatric Interview (M.I.N.I.): the development and validation of a structured diagnostic psychiatric interview for DSM-IV and ICD-10. *J Clin Psychiatry* **59** Suppl 20, 22–33; quiz 34–57 (1998). 9881538

[8] Kadouri, A., Corruble, E. & Falissard, B. The improved Clinical Global Impression Scale (iCGI): development and validation in depression. *BMC Psychiatry* **7**, 7, doi:1471-244X-7-7 [pii] 10.1186/ (2007). 10.1186/1471-244X-7-7 PMC1802073 17284321

[9] Beck AT, Ward CH, Mendelson M, Mock J, Erbaugh J (1961). An inventory for measuring depression. Arch Gen Psychiatry.

[10] Litte JC, McPhail NI (1973). Measures of depressive mood at monthly intervals. Br J Psychiatry.

[11] McCormack HM, Horne DJ, Sheather S (1988). Clinical applications of visual analogue scales: a critical review. Psychol Med.

[12] Williams, V. S., Morlock, R. J. & Feltner, D. Psychometric evaluation of a visual analog scale for the assessment of anxiety. *Health Qual Life Outcomes* **8**, 57, doi:1477-7525-8-57 [pii] 10.1186/ (2010). 10.1186/1477-7525-8-57 PMC2904728 20529361

[13] Shrout PE, Fleiss JL (1979). Intraclass correlations: uses in assessing rater reliability. Psychol Bull.

[14] Maechler DBaM (2009). lme4: Linear mixed-effects models using S4 classes. R package version.

[15] Naudet, F., Millet, B., Reymann, J. M. & Falissard, B. Improving study design for antidepressant effectiveness assessment. *Int J Methods Psychiatr Res* doi:10.1002/mpr.1391 (2013). 10.1002/mpr.1391 PMC6878496 24038333

[16] Cicchetti DV (1994). Guidelines, criteria, and rules of thumb for evaluating normed and standardized assessment instruments in psychology. Psychological assessment.

[17] Rabkin, J. G. *et al*. How blind is blind? Assessment of patient and doctor medication guesses in a placebo-controlled trial of imipramine and phenelzine. *Psychiatry Res* **19**, 75–86, doi:0165-1781(86)90094-6[pii] (1986). 10.1016/0165-1781(86)90094-6 3538107

[18] Perlis, R. H. *et al*. Assuring That Double-Blind Is Blind. *Am J Psychiatry* **167**, 250–252, doi:167/3/250 [pii] 10.1176/appi.ajp.2009.09060820 (2010). 10.1176/appi.ajp.2009.09060820 20194487

[19] Naudet F, Maria AS, Falissard B (2011). Antidepressant Response in Major Depressive Disorder: A Meta-Regression Comparison of Randomized Controlled Trials and Observational Studies. PLoS One.

[20] Rutherford, B., Sneed, J., Devanand, D., Eisenstadt, R. & Roose, S. Antidepressant study design affects patient expectancy: a pilot study. *Psychol Med* **40**, 781–788, doi:S0033291709991085[pii] 10.1017/S0033291709991085 (2010). 10.1017/S0033291709991085 PMC3784014 19732481

[21] Rutherford, B. R., Sneed, J. R. & Roose, S. P. Does study design influence outcome? The effects of placebo control and treatment duration in antidepressant trials. *Psychother Psychosom* **78**, 172–181, doi:000209348 [pii] 10.1159/000209348 (2009). 10.1159/000209348 PMC3785090 19321970

[22] Sinyor, M. *et al*. Does inclusion of a placebo arm influence response to active antidepressant treatment in randomized controlled trials? Results from pooled and meta-analyses. *J Clin Psychiatry* **71**, 270–279, doi:10.4088/JCP.08r04516blu (2010). 10.4088/JCP.08r04516blu 20122371

[23] Sneed JR (2008). Design makes a difference: a meta-analysis of antidepressant response rates in placebo-controlled versus comparator trials in late-life depression. Am J Geriatr Psychiatry.

[24] Papakostas GI, Fava M (2009). Does the probability of receiving placebo influence clinical trial outcome? A meta-regression of double-blind, randomized clinical trials in MDD. Eur Neuropsychopharmacol.

[25] Krendl, A. C., Kensinger, E. A. & Ambady, N. How does the brain regulate negative bias to stigma? *Soc Cogn Affect Neurosci* **7**, 715–726, doi:nsr046 [pii] 10.1093/scan/nsr046 (2012). 10.1093/scan/nsr046 PMC3427867 21896496

[26] Henderson, C. *et al*. Mental health-related stigma in health care and mental health-care settings. *Lancet Psychiatry* **1**, 467–482, doi:S2215-0366(14)00023-6 [pii] 10.1016/S2215-0366(14)00023-6 (2014). 10.1016/S2215-0366(14)00023-6 26361202

[27] Gray JE, Higenbottam JA (1982). The pyjama game: placement in pyjamas in Canadian psychiatric hospitals. Can Ment Health.

[28] Walker VJ, Voineskos G, Dunleavy DL (1971). The effects of psychiatric nurses ceasing to wear uniform. Br J Psychiatry.

[29] Naudet, F. *et al*. Understanding the Antidepressant Debate in the Treatment of Major Depressive Disorder. *Therapie* **70**, 321–327, doi:10.2515/therapie/2014228 therapie140028 [pii] (2015). 10.2515/therapie/2014228 25679188

[30] Moncrieff, J. & Kirsch, I. Efficacy of antidepressants in adults. *Bmj* **331**, 155-157, doi:331/7509/155 [pii] 10.1136/bmj.331.7509.155 (2005). 10.1136/bmj.331.7509.155 PMC558707 16020858

[31] Moncrieff, J. & Kirsch, I. Empirically derived criteria cast doubt on the clinical significance of antidepressant-placebo differences. *Contemp Clin Trials* **43**, 60-62, doi:S1551-7144(15)30003-3 [pii] 10.1016/j.cct.2015.05.005 (2015). 10.1016/j.cct.2015.05.005 25979317

[32] Thase, M. E., Mahableshwarkar, A. R., Dragheim, M., Loft, H. & Vieta, E. A meta-analysis of randomized, placebo-controlled trials of vortioxetine for the treatment of major depressive disorder in adults. *Eur Neuropsychopharmacol* **26**, 979–993, doi:S0924-977X(16)30005-0 [pii] 10.1016/j.euroneuro.2016.03.007 (2016). 10.1016/j.euroneuro.2016.03.007 27139079

[33] Rosenhan DL (1973). On being sane in insane places. Science.

[34] Moncrieff, J. The creation of the concept of an antidepressant: an historical analysis. *Soc Sci Med* **66**, 2346–2355, doi:S0277-9536(08)00012-9 [pii] 10.1016/j.socscimed.2008.01.047 (2008). 10.1016/j.socscimed.2008.01.047 18321627

[35] Pilgrim D, Bentall R (1999). The medicalisation of misery: A critical realist analysis of the concept of depression. Journal of mental health.

